# Blood Bonds: Transforming Blood Donation Through Innovation, Inclusion, and Engagement

**DOI:** 10.2196/63817

**Published:** 2024-09-27

**Authors:** Ankita Sagar

**Affiliations:** 1 Creighton University School of Medicine CommonSpirit Health Monmouth Junction, NJ United States

**Keywords:** blood donor, engagement, digital health, health technology, EBM, inclusive, inclusivity, shared decision-making, evidence-based medicine, blood shortage, blood product, transfusion, transfusions, donor, donation, hematology, perioperative medicine, surgery

## Abstract

The journey of receiving blood as a patient with transfusion-dependent beta thalassemia has profoundly shaped my understanding of the life-saving power of blood donation. This personal experience underscores the critical importance of blood donors, not just for individual recipients but for the broader community, enhancing public health, productivity, and well-being. There are several challenges to securing a blood donor pool in current health care climate. Solutions that focus on the engagement of donors, clinicians, and patients are key to improving the donor pool and utilizing the blood supply in a judicious manner.

## Background

The journey of receiving 771 units of blood as a patient with transfusion-dependent beta thalassemia has profoundly shaped my perspective on the life-saving generosity of blood donors. The altruism of my blood donors has not only sustained my life to reach my 40th birthday but also serves as a poignant reminder of human compassion in our world for me, giving me hope as both a physician and a patient.

Beyond individual recipients, blood donors contribute to community-wide benefits, enhancing health, longevity, and productivity across diverse professions and lifestyles. Blood products represent a critical facet of medical treatment that has yet to be effectively revolutionized on a large scale. Despite advancements in various medical technologies and therapies, the fundamental process of blood donation and transfusion remains reliant on the altruism of donors. Despite the substantial volume of blood donated in the United States—13.6 million units annually—the supply remains precarious, with 25% of community blood centers reporting only 1 day or less of blood supply via America’s Blood Centers [1,2]. The limited supply is a reflection of the changing dynamics and profiles of blood donors: donations from adults aged 18 to 25 years declined by 32%, while those from adults aged 25-64 years and 65 years or older increased by 14% and 41%, respectively [3]. This decline in younger donors signals a concerning trend, indicating potential challenges in engaging and retaining future blood donors, which could exacerbate supply issues in the years to come. This illustrates the critical challenges in securing blood supply within the United States. Addressing these challenges demands a coordinated effort to increase donations and optimize the use of blood products, involving stakeholders such as patients, clinical teams, and donors in a strategic framework aimed at securing a sustainable blood supply for all who depend on it.

## Engaging Clinical Teams

Access to established guidelines or frameworks pertaining to blood product utilization can significantly enhance engagement among clinical teams and patients. Recent studies have identified considerable variability in intraoperative blood product administration guidelines across professional societies, particularly concerning indications, decision-making processes, and the underlying evidence base [4-6]. This underscores a critical need for dedicated research into outcomes related to intraoperative and postoperative blood product use, with the aim of establishing standardized clinical guidelines.

Transfusion remains one of the most frequent procedures in surgical settings, with efforts focused on improving the judicious use of red blood cell products for surgical patients [7]. Despite initiatives such as updated clinical guidelines being available, implementation in the surgical continuum has been gradual, reflecting persistent self-identified knowledge gaps among physicians and advanced practice providers [7-9]. Furthermore, clinicians recognize the importance and applicability of transfusion medicine knowledge to their daily clinical practice and welcome further training and education [9]. Moreover, when a self-identified need is addressed via targeted educational interventions and implementation of an evidence-informed guideline, a positive impact on knowledge outcomes, patient outcomes, and enhancement in practice is often noted [10].

The use of technology offers promising methods to enhance clinician engagement and decision-making. Augmented intelligence and clinical decision support tools allow machine learning to assist in predicting bleeding risk and optimizing transfusion strategies, especially in settings such as treating gastrointestinal bleeding in the intensive care unit [11]. These and other advancements underscore the transformative potential of technology in augmenting clinical practice and improving patient outcomes [12]. Nevertheless, prior to vast use of such technology, potential harms caused by and biases within the algorithm of such technology needs to be further studied with due diligence [7].

## Engaging Patients

Among patients requiring blood products, shared decision-making and incorporating patients’ preferences and values are pivotal in determining individualized transfusion thresholds [13]. Interdisciplinary clinical teams regularly engage in shared decision-making processes that balance patient preferences and evidence-informed clinical protocols. This collaborative approach has proven successful in areas such as cancer prevention and screening, advance directive planning, statin therapy for primary prevention of coronary artery disease, and immunotherapy in oncology. By adopting similar strategies for transfusion needs, individualized plans for anticipated transfusion needs may successfully align with patients’ values and clinical science to ensure the judicious use of blood products.

## Engaging Blood Donors

Broadening the donor pool and enhancing retention efforts are essential to mitigating blood shortages. Revisiting restrictive policies, such as the Food and Drug Administration’s recent lift on blood donation restrictions for LGBTQ+ (lesbian, gay, bisexual, transgender, queer) individuals in alignment with global scientific evidence and inclusive policies, promotes equity and significantly bolsters donor numbers [14]. Similarly, revisiting strategies to engaging younger generations in blood donation requires authentic, relatable approaches that highlight its altruistic impact. Engaging campaigns emphasizing the immediate life-saving potential of donations, rather than monetary incentives, resonate more deeply with younger donors who prioritize values and community impact [15]. Furthermore, combining campaigns with personal narratives from donors and recipients can further inspire community dialogue and encourage new and returning donors, reinforcing the vital role of blood donation in saving lives and fostering community solidarity [16].

Recent innovations, like virtual reality experiences during blood donation, have shown promise in enhancing donor engagement through positive emotional experiences. Studies have shown that involving mixed reality, a form of virtual reality, reduces donor anxiety and enhances satisfaction, making blood donation a mindful and positive experience [17]. Such applications are actively being employed given their transformative impact, by bolstering blood bank efforts or donor recruitment [18,19].

Technology and scientific advances can help ensure the sustainability of blood donation for years to come. Blood donors provide life-sustaining treatment for transfusion-dependent patients like me, and their significant contributions deserve the utmost commitment to ensuring a reliable blood supply. By leveraging evidence-based guidelines to optimize blood product utilization, fostering shared decision-making, enhancing clinical team engagement, and embracing impactful donor engagement strategies, we can effectively mitigate future blood shortages and secure a sustainable future for transfusion medicine. As both a patient and physician, I am hopeful about the potential of these advancements in ensuring that every person in need can rely on the life-saving generosity of blood donors.

## Abbreviations

LGBTQ+: lesbian, gay, bisexual, transgender, queer
